# Cutting costs of multiple mini-interviews – changes in reliability and efficiency of the Hamburg medical school admission test between two applications

**DOI:** 10.1186/1472-6920-14-54

**Published:** 2014-03-19

**Authors:** Johanna C Hissbach, Susanne Sehner, Sigrid Harendza, Wolfgang Hampe

**Affiliations:** 1 Department of Biochemistry and Molecular Cell Biology, Center for Experimental Medicine, University Medical Center Hamburg-Eppendorf, Martinistraße 52, Hamburg 20246, Germany; 2 Department of Medical Biometry and Epidemiology, Center for Experimental Medicine, University Medical Center Hamburg-Eppendorf, Martinistraße 52, Hamburg 20246, Germany; 3 III Department of Internal Medicine, Center for Internal Medicine, University Medical Center Hamburg-Eppendorf, Martinistraße 52, Hamburg 20246, Germany

**Keywords:** Multiple mini interview, Cost-effectiveness analysis, Reliability, Optimization

## Abstract

**Background:**

Multiple mini-interviews (MMIs) are a valuable tool in medical school selection due to their broad acceptance and promising psychometric properties. With respect to the high expenses associated with this procedure, the discussion about its feasibility should be extended to cost-effectiveness issues.

**Methods:**

Following a pilot test of MMIs for medical school admission at Hamburg University in 2009 (HAM-Int), we took several actions to improve reliability and to reduce costs of the subsequent procedure in 2010. For both years, we assessed overall and inter-rater reliabilities based on multilevel analyses. Moreover, we provide a detailed specification of costs, as well as an extrapolation of the interrelation of costs, reliability, and the setup of the procedure.

**Results:**

The overall reliability of the initial 2009 HAM-Int procedure with twelve stations and an average of 2.33 raters per station was ICC=0.75. Following the improvement actions, in 2010 the ICC remained stable at 0.76, despite the reduction of the process to nine stations and 2.17 raters per station. Moreover, costs were cut down from $915 to $495 per candidate. With the 2010 modalities, we could have reached an ICC of 0.80 with 16 single rater stations ($570 per candidate).

**Conclusions:**

With respect to reliability and cost-efficiency, it is generally worthwhile to invest in scoring, rater training and scenario development. Moreover, it is more beneficial to increase the number of stations instead of raters within stations. However, if we want to achieve more than 80 % reliability, a minor improvement is paid with skyrocketing costs.

## Background

Admission to medical school is a field of feisty debate. Usually, measures of academic achievement and interview performance are used for admission decisions. Assets and drawbacks of these different approaches allude to psychometric properties and costs. School grades such as grade point average (GPA) and high stakes ability tests are usually easily administered, cost efficient and psychometrically sound but they disregard personality factors that might be crucial for a medical career (e.g. [1-3]). On the other hand, interviews have high face validity [4], but evidence for the reliability and validity of panel interviews is scarce.

The multiple mini-interview (MMI) with its multiple sampling approach is widely accepted by raters and candidates [5-7], and it is regarded as a comparatively reliable measure of non-cognitive skills [8]. However, reliability coefficients vary substantially depending on the target population, setting variables, study design, and methods used, which impedes the comparison of results. In undergraduate medical school selection, reliability measures obtained on the basis of generalizability method [9] ranged from 0.63 to 0.79 [10-13]. Most coefficients for nine station procedures with one or two observers per station lie around G=0.75.

Another concern specifically addresses the cost-effectiveness of MMI. The costs and the effort of faculty are essential for officials to refrain from introducing MMIs [10]. The expenses associated with such a procedure depend mainly on varying modalities of the process. Even though there is evidence that MMIs are more cost-effective than traditional panel interviews [6,14,15], costs are still high as compared to paper and pencil tests. Eva et al. report the costs of the actual process on the interview day (about $35 per candidate) but do not include the costs generated in the framework of project preparation and organization [6]. Rosenfeld et al. provided an overview of the time requirements for mounting multiple mini-interviews and traditional interviews [14]. To interview 400 candidates with the MMI procedure they calculated a maximum of 1,078 staff hours (278 staff hours for the organization and 800 observer hours). Additional costs of $5,440 arose from the creation of stations ($50 per station for three hours creation time), infrastructure, and miscellaneous expenses. If we assume an average hourly rate of $50 for their staff, then the total costs would be approximately $150 per candidate.

In Tel-Aviv, Ziv et al. developed a medical school admission tool with MMI concepts (MOR) and found the inter-rater reliability of the behavioral interview stations was moderate [16]. The total cost of MOR process was approximately $300 per candidate but further information on the existing costs has not been provided.

In another study, costs of an Australian MMI procedure from 2009 were roughly AU $450 per candidate [17] – the costs reported, however, were mostly on candidates’ side, with airfares being the major factor.

### Student selection at Hamburg medical school

In the 1990s, Hamburg Medical School conducted unstructured interviews for admission. Many faculty members were dissatisfied with this procedure, and the interviews were stopped within the scope of a change in federal law. With the introduction of a test in natural sciences for student admission in 2008 [18,19], the significance of psychosocial skills came to the fore. In March 2009, the faculty board decided to adopt the MMI format for a pilot test with a small number of candidates, aiming for a stepwise selection procedure in 2010: The GPA and HAM-Nat scores were applied to preselect candidates whose psychosocial skills were then assessed by the HAM-Int (“**H**amburg **A**ssessment Test for **M**edicine - Interview”).

### The HAM-Int pilot (2009)

In a survey among the heads of clinical departments and members of the curriculum committees the following eight psychosocial characteristics received the highest ratings: integrity, self-reflection, empathy, self-regulation, stress resistance, decision-making abilities, respect, and motivation to study medicine. The participants of a faculty development workshop wrote the MMI scenarios, keeping the specified psychosocial skills in mind. These drafts were later discussed with psychologists and educational researchers and thereupon modified or rejected. Some of the defined skills were wide ranging or could not to be validly tested (e.g. integrity). Therefore, it was impossible to achieve a word-for-word translation of scenario characteristics. In total, twelve five-minute stations were assembled for the 2009 circuit.

We found a relatively low overall reliability coefficient (ICC=0.75 for twelve stations and a mean of 2.3 raters per station) as compared to those reported in other studies [20]. This raised the question as to which actions would enhance the reliability of the multiple mini-interview. Uijtdehaage et al. [21] found that a few changes in the procedure improved the reliability from G=0.59 to G=0.71. The increase in reliability was mainly due to a rise in candidate variation. The authors argue that maybe the change of venue – such as interviews were conducted in a different building – made the procedure less intimidating and therefore less stressful for candidates.

The feedback of raters and candidates drew our attention to the parameters, i.e. scenarios, score sheets, and rater training, aimed at improving reliability. We compare the results from the 2009 pilot test and the 2010 procedure.

This paper focuses on two aspects of MMI improvement: fine-tuning and cost-effectiveness. Our research questions were: Did our actions to improve the procedure enhance overall reliability? Which is the most efficient and practicable way to reach satisfactory reliability?

## Methods

### Candidates

In 2009, applicants for Hamburg Medical School were asked to state if they preferred to take the HAM-Nat test or the HAM-Int. We used the HAM-Int pilot to award 30 university places on the basis of interview results (in combination with GPA). The remaining places were allocated by HAM-Nat results (in combination with GPA). Among the 215 applicants who preferred the interviews to the HAM-Nat test, those 80 with the highest GPA were invited. The others were assigned to the HAM-Nat test. In 2010, we felt prepared to test 200 candidates who were preselected by the HAM-Nat test and GPA. All candidates took the HAM-Nat test, and those with excellent GPA and HAM-Nat scores (rank 1–100) were admitted without further testing, while the next 200 were invited to take the interviews. One hundred and fifteen further places were available. All candidates gave written informed consent.

### Procedure

All interviews of one year took place on a single day in parallel circuits and consecutive rounds. Interviewers remained at their station during the day. Candidates were randomly assigned to circuit and round. In 2010, the number of circuits was increased from two to four and the number of rounds from three to five. To preclude a leak of scenario contents, all candidates checked in at the same time in the morning in 2009. As candidates perceived the waiting period before the start of the interviews as being quite stressful, in 2010 all candidates checked in just before they started their interview cycle. We also provided the raters with personalized score sheets in order of appearance of candidates, which substantially improved the interview cycle. An overview of the changes made to the procedure is given in Table 1.

**Table 1:** Changes made to the procedure (2009 – 2010)

	**2009**	**2010**
Number of target variables	8	3
Number of stations	12	9
Duration of interview cycle (minutes)	78	58.5
Number of rounds	4	5
Number of circuits	2	4
Average ratings per candidate and station	2.3	2.17
Number of ratings per candidate	28	19.5
Number of candidates	80	200
Rater training hours	2	4
Interviewers give ratings	Yes	No
Score sheets	No anchoring	Anchoring, personalization
Check-in time on interview day	Simultaneous	Variable

### Stations

In 2009, twelve five-minute stations with 1.5 minutes change-over time were assembled. Actors experienced with objective structured clinical examinations (OSCEs) from the in-house simulated patients program were trained for six scenarios. We provided prompting questions for the interviewers for the other six stations.

As it had turned out to be challenging to write scenarios which reflected the eight different target variables, the steering committee decided to focus on a core set of three in 2010: empathy, communication skills, and self-regulation. In 2010, nine five-minute stations were assembled. Those four stations that appeared to have worked best in 2009 were refined and reused, and five new stations were developed with more time and effort spent into testing and revision. In total, five stations involved actors.

### Score sheets

The 2009 scoring sheets comprised three specific items and one global rating on a 6-point Likert scale. The numerically anchored scale ranged from 0–5 points. The specific items reflected e.g. communication skills, the formal presentation of a problem, empathy or respect in a social interaction, depending on the main focus of the station. The global rating was meant to reflect overall performance, including aspects not covered by the specific items. As the two lowest categories were only used in less than 5% of the global ratings, we changed the scale to a verbally anchored, 5 point-Likert scale in 2010. The scale ranged from 1 (very poor) to 5 (very good). In a thorough revision of all score sheets, we included detailed descriptions of unwanted and desired candidate behavior as anchors at three points along the scale (very poor performance, mediocre performance and very good performance). Raters were encouraged to use the full range of scores.

### Raters and rater training

Hospital staff volunteered to take part in the interviews. Raters were released from work for the interview day within the scope of their regular contracts to be involved in the process. Mixed-gender rater teams of at least one professional from the psychosocial department and one experienced clinician were randomly assigned to stations to include a broad spectrum of judgments. The rationale to do so originated from the fact that not all candidates encountered the same set of interviewers. We aimed to ensure that all candidates saw an equal number of men and women as well as of psychologists and physicians.

All raters received a general instruction to familiarize them with the MMI procedure. They were then grouped within their specific stations, discussed their scenario, and had several practice runs with simulated candidates (students) to standardize scoring between the parallel circuits. While in 2009 the rater training session of two hours was held just before interviews started, the training was extended to a four hour session on the day preceding the interviews in 2010. While in 2009 interviewers rated the candidates’ performance, we refrained from this practice in the following year as a result of the interviewers’ feedback. They stated that is was too demanding to interview and to give a reliable rating at the same time.

### Statistical analysis

Due to the naturalistic setting we have a partially crossed and nested design. Different sources of variability were estimated by means of a random intercept model with restricted maximum likelihood (REML) method. All analyses were conducted using IBM SPSS Statistics, Version 19.0.0 (2010).

As each candidate encountered all twelve or nine stations, respectively, candidates were fully crossed with stations but nested within circuit. Raters were nested within station and circuit as each rater was trained for one specific station. We constructed two different models. In the first model we examined the different sources of variability (random intercepts): candidate, station, rater, and candidate*station. The candidate effect reflects systematic differences in performance between candidates. The station effect represents systematic differences in station difficulty, while the candidate*station effect accounts for differences in the way candidates coped with the different stations. This effect is non-systematic and reflects a candidate specific profile of strengths and weaknesses with regard to stations. As raters remained at their station throughout the test, systematic differences in stringency (rater effect) could be estimated, while the rater*candidate effect (rater candidate taste) could not be separated from error. We apportioned all remaining variance to this term.

Corresponding to Generalizability Theory [22] we determined sources of measurement error by means of a multilevel random intercept model [23]. We took the ICCs as a G-coefficient for relative decisions as we included only those terms that affect the rank ordering of candidates. The reliability of the procedure is the proportion of variance attributable to candidates to total variance. As candidates were assigned to different sets of raters, systematic differences in rater stringency can have an effect on the ranking of candidates. Therefore, we adjusted for rater stringency as proposed by Roberts et al. [24] by including a fixed rater effect.

Unwanted sources of variability are due to the candidate specific station differences (V_cand*stat_), namely candidate station taste, while systematic differences in station difficulty have no effect on the rank order, as all candidates encountered the same stations. All remaining residual variance was attributed to rater candidate taste (V_cand*rater_). The following formula was used for the calculation of the overall reliability:

$$\mathrm{Rel}=V_{\mathrm{cand}}/\left(V_{\mathrm{cand}}+V_{\mathrm{cand}*\mathrm{stat}}/n_{\mathrm{stat}}+V_{\mathrm{cand}*\mathrm{rater}}/n_{\mathrm{rater}}\right)$$

As a measure of inter-rater reliabilities (IRR) in the different stations we report intraclass correlations (ICC) for average measures (consistency) with two-way random effects.

## Results

### Descriptive statistics

Candidate and rater characteristics are displayed in Table 2. As the correlation of the global score and the mean score of the three specific items was 0.93 (95% CI: 0.92; 0.94) in 2009, we used the global score for all analyses. In 2009, the lowest two categories (0 and 1) of the scale were used in less than five percent which was also true for the lowest category (1) in 2010. Practically, this resulted in a four point scale for both years. The mean difficulties and item-total correlations on station level as well as interrater reliability measures (ICC, average measures) are given in Table 3. The mean inter-station correlation in 2009 was 0.20 (min: -0.11; max: 0.44) and 0.19 in 2010 (min: 0.07; max: 0.32).

**Table 2:** Descriptive statistics of candidates and raters

	**2009**		**2010**	
	**N (%)**			
Raters	57		94	
Females	23	(40%)	50	(53%)
Candidates invited	80		200	
Candidates tested	78	(98%)	193	(97%)
Females	50	(64%)	112	(58%)
German	71	(91%)	185	(96%)
EU	2	(3%)	5	(2%)
Non-EU	5	(6%)	3	(2%)
	**Mean (sd)**			
Age	20.2	(4.1)	19.8	(1.6)
GPA (highschool)	1.7	(0.2)	1.7	(0.2)
% achieved of max. HAM-Int score	68.7		64.7	
Mean station result	3.43^a^	(0.83)	3.23^b^	(1.05)

^a^scale from 0–5; ^b^scale from 1–5.

**Table 3:** Station characteristics

	**Station description**	**Mean station difficulty (sd)**				**Corrected item total correlation**		**IRR (ICC)**	
		**2009**^**a**^		**2010**^**b**^		**2009**^**a**^	**2010**^**b**^	**2009**^**a**^	**2010**^**b**^
1	Personal dilemma I (SP)	3.97	(0.97)			0.51		0.42	
2	Empathetic communication I (SP)	3.33	(1.04)			0.42		0.75	
3	Conflict resolution I (SP)	3.37	(1.08)			0.36		0.78	
4	Motivation interview I	3.50	(1.09)			0.48		0.58	
5	Ethical dilemma I	3.17	(0.93)			0.37		0.65	
6	Evaluation social interaction	3.51	(1.01)			0.40		0.65	
7	Conflict resolution II (SP)	3.27	(0.99)			0.10		0.44	
8	Decision making	3.72	(0.85)			0.30		0.50	
9	Conflict resolution II	3.29	(0.86)	3.36	(1.02)	0.42	0.35	0.77	0.71
10	Breaking bad news (SP)	3.80	(0.89)	2.92	(1.07)	0.61	0.40	0.70	0.84
11	Clarification of a situation (SP)	3.18	(1.04)	3.09	(1.13)	0.33	0.38	0.73	0.57
12	Ethical dilemma II	3.01	(1.21)	3.22	(0.98)	0.31	0.27	0.74	0.78
13	Conflict resolution III (SP)			3.36	(1.02)		0.45		0.55
14	Empathetic communication II (SP)			3.19	(0.98)		0.36		0.83
15	Decision making II			2.96	(0.97)		0.37		0.78
16	Conflict resolution III (SP)			3.29	(1.24)		0.41		0.64
17	Motivation interview II			3.68	(1.02)		0.22		0.61
	Mean all stations	3.43	(0.83)	3.23	(1.05)	0.38	0.36		
	All two-rater stations							0.68	0.71
	All three-rater stations							0.67	0.80

^a^scale from 0–5; ^b^scale from 1–5.

### Estimation of variance components (model 1)

In 2009, twelve percent of the total variance was attributable to the variability between candidates, and roughly one third of the total variability resulted from differences in candidate performance in the different stations. More than half of the total variability (56%) was accounted for by varying stringency of raters (8%), as well as rater candidate taste and error (48%). Systematic differences in station difficulty only accounted for five percent of the total variability. This was the only insignificant effect (p=0.114).

With the 2010 procedure, we found a rise in the variance attributable to candidate (18%) and candidate*station (33%), while differences in rater behaviour declined (45%). The station effect remained insignificant (p=0.100). All variance components and confidence intervals are displayed in Table 4.

**Table 4:** Variance components of model 1 (without adjustment for rater stringency)

		**2009**		**2010**	
**Effect**		**Variance components (95% CI)**	**%**	**Variance components (95% CI)**	**%**
Cand	Systematic differences in candidate performance	0.13 (0.09-0.20)	12.2	0.21 (0.16-0.27)	18.1
Stat	Systematic differences in station difficulty	0.06 (0.02-0.19)	5.0	0.04 (0.01-0.13)	3.3
Rater	Systematic differences in rater stringency	0.09 (0.05-0.14)	7.8	0.04 (0.03-0.07)	3.8
Cand*stat	Differences in candidate performance between stations	0.30 (0.25-0.36)	27.6	0.39 (0.34-0.43)	33.3
Rater*cand (+ error)	Differences in rater candidate taste, and residual variance	0.52 (0.48-0.56)	47.5	0.48 (0.45-0.51)	41.5
Total		1.09	100	1.16	100

Rel = V_cand._/(V_cand_ + V_stat_/n_stat_ + V_rater_/n_ratings per cand_ + V_cand*stat_/n_stat_ + V_rater*cand_/n_ratings per cand_).

2009: Rel = V_cand_/(V_cand_ + V_stat_/12 + V_rater_/28 + V_cand*stat_/12 + V_rater*cand_/28) = 0.72.

2010: Rel = V_cand_/(V_cand_ + V_stat_/9 + V_rater_/19.5 + V_cand*stat_/9 + V_rater*cand_/19.5) = 0.74.

### Estimation of overall reliability (model 2)

We used the second model to compute candidates’ total scores which were adjusted for rater stringency. The variance components are displayed in Table 5. Overall reliability of the pilot test was ICC=0.75.

**Table 5:** Variance components of model 2 (reliability model with adjustment for rater stringency)

		**2009**		**2010**	
**Effect**		**Variance components (95% CI)**	**%**	**Variance components (95% CI)**	**%**
Cand	Systematic differences in candidate performance	0.13 (0.08-0.20)	13.9	0.21 (0.16-0.28)	19.6
Cand*stat	Differences in candidate performance between stations	0.30 (0.02-0.19)	31.6	0.38 (0.34-0.43)	35.6
Rater*cand (+ error)	Adjusted differences in rater candidate taste, and residual variance	0.52 (0.55-0.64)	54.5	0.48 (0.45-0.51)	44.8
Total		0.95	100	1.07	100

2009: Rel = V_cand_/(V_cand_ + V_cand*stat_/12 + V_rater*cand_/28) = 0.75.

2010: Rel = V_cand_/(V_cand_ + V_cand*station_/9 + V_rater*cand_/19.5) = 0.76.

**Figure 1:**
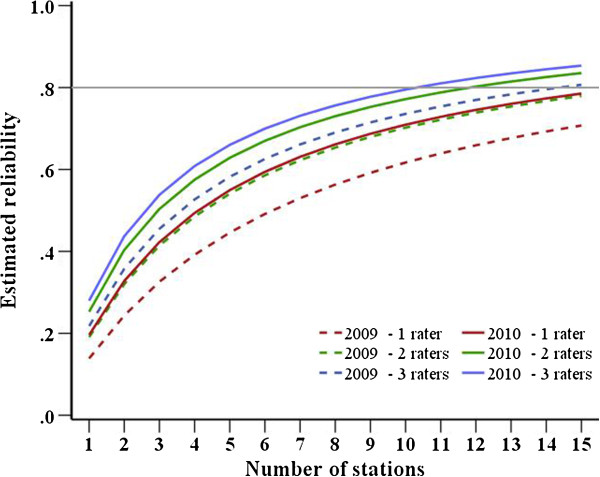
Estimation of total test reliability with different numbers of raters and stations (2009 vs. 2010).

In 2010, overall reliability was ICC=0.76. Figure 1 illustrates which amount of reliability is to expect if we vary the number of stations and raters per station while keeping all other conditions constant. With eight stations and two raters per station, the overall reliability would have increased from 65% to 73% between 2009 and 2010.

### Costs

Expenses for the HAM-Int mainly arose from the working hours required for station development and the interview day itself. For a detailed description of costs see Tables 6 and 7. The education building at Hamburg Medical School offers enough adjacent rooms to conduct nine stations on four levels. We did not include facility costs as we were not charged by the faculty.

**Table 6:** Costs of the 2009 procedure

**2009 procedure**	**Scientist (50 $/h)**		**Admin staff (30 $/h)**		**Actor (10 $/h)**		**Student (13 $/h)**		**Material**	**Total**
	**Hours**	**$**	**Hours**	**$**	**Hours**	**$**	**Hours**	**$**	**$**	**$**
Writing and adaptation of 12 scenarios and score sheets	475	23,750								**23,750**
Organization of test runs			24	720						**720**
Test runs	180	9,000	24	720	36	360	36	468	1,500	**12,048**
**Total costs for development of 12 stations**	**655**	**32,750**	**48**	**1,440**	**36**	**360**	**36**	**468**	**1,500**	**36,518**
Room acquisition			3	90						**90**
Rater recruitment and allocation	3	150	15	450						**600**
Training of actors	50	2,500			150	1,500				**4,000**
Invitation of / feedback to candidates			5	150						**150**
Printing of score sheets			5	150			10	130	200	**480**
Preparation and clearance of rooms	5	250	10	300			25	325		**875**
Rater training and testing of candidates	480	24,000	25	750	150	1,500	120	1,560		**27,810**
Catering for raters									1,500	**1,500**
Scanning of score sheets and data analysis	10	500	20	600						**1,100**
**Total costs for implementation**	**548**	**27,400**	**83**	**2,490**	**300**	**3,000**	**155**	**2,015**	**1,500**	**36,405**
Additional costs for first implementation	100	5,000	100	3,000			50	650	1,500	**10,150**
**Total costs for 80 candidates**	**1,303**	**65,150**	**231**	**6,930**	**336**	**3,360**	**241**	**3,133**	**4,700**	**83,073**

**Table 7:** Costs of the 2010 procedure

**2010 procedure**	**Scientist**		**Admin staff**		**Actor**		**Student**		**Material**	**Total**	**Average costs ($)**		**Fixed costs ($)**
	**(50 $/h)**		**(30 $/h)**		**(10 $/h)**		**(13 $/h)**						
	**Hours**	**$**	**Hours**	**$**	**Hours**	**$**	**Hours**	**$**	**$**	**$**	**Station**	**Rater**	**Overall**
Adaptation of 4 scenarios and score sheets	75	3,750								**3,750**			
Writing of 5 new scenarios and score sheets	200	10,000								**10,000**			
Organization of test runs			4	120						**120**			
Test runs	80	4,000	8	240	10	100	25	325	400	**5,065**			
**Total costs for development of 9 stations**	**355**	**17,750**	**12**	**360**	**10**	**100**	**25**	**325**	**400**	**18,935**	2,104^a^		
Room acquisition			10	300						**300**			300
Rater recruitment and allocation	3	150	20	600						**750**			750
Training of actors	30	1,500			150	1,500				**3,000**	222^a^		1,000
Invitation of / feedback to candidates			15	450						**450**			450
Printing of score sheets			10	300			20	260	700	**1,260**			1,260
Preparation and clearance of rooms	5	250	10	300			30	390		**940**			940
Rater training and testing of candidates	995	49,750	50	1,500	400	4,000	200	2,600		**57,850**		2,551^b^	8,100
Interviewer training and testing of candidates	205	10,250								**10,250**	1,139^a^		
Catering for raters									4000	**4,000**		170^b^	680
Scanning of score sheets and data analysis	10	500	20	600						**1,100**			1,100
**Total costs for implementation**	**1,248**	**62,400**	**135**	**4,050**	**550**	**5,500**	**250**	**3,250**	**4,700**	**79,900**			
**Total costs for 200 candidates**	**1,603**	**80,150**	**147**	**4,410**	**560**	**5,600**	**275**	**3,575**	**5,100**	**98,835**	3,465	2,721	14,581

^a^average costs per station (costs divided by the number of stations (9)); ^b^average costs per rater (costs divided by the number of raters per candidate (19.5)).

For the 2009 run we minimized development time and effort by adapting some ideas from published scenarios. Generally, two psychologists or physicians devised a scenario and drafted scoring sheets and detailed instructions for the actors or interviewers. It was especially time consuming to establish clear guidelines for performance scoring to enhance standardisation of the ratings. We conducted the first HAM-Int with relatively few candidates to gain experience for the following years. The total costs were roughly $73,100 plus $10,150 additional costs for the first implementation, i.e. $1040 per candidate ($915 without additional costs). The total costs per candidate were cut to $495 in 2010. The largest reduction of costs was related to station development costs which were almost halved from $36,600 to $18,900.

**Figure 2:**
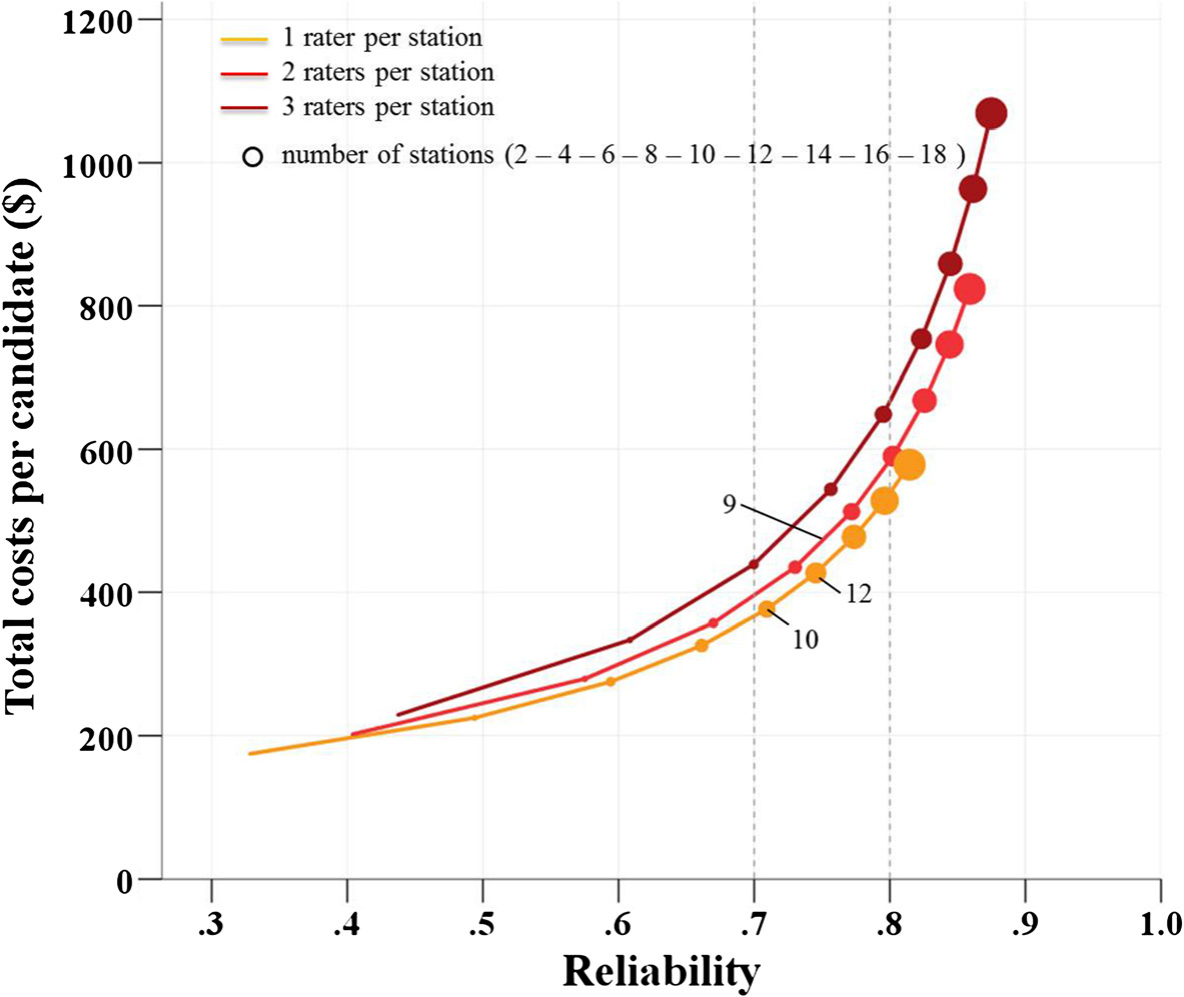
Interrelation of costs and reliability (2010 HAM-Int procedure).

Figure 2 depicts the relation of costs and reliability on the premises of the 2010 procedure. We divided station and rater costs into a fixed part and an averaged part (Table 7). The fixed expenses for the whole procedure ($14,580) arise irrespectively of the number of stations and raters used while the average costs per station ($3,465) and the average costs per rater ($2,721) are added depending on the comprehensiveness of the procedure. We are now able to extrapolate what would happen to the costs and to the reliability if we varied the number of stations and raters. This calculation is based on the premise that interviewers do not give ratings, and that the extension of the procedure by more stations relies on on a 1:1 ratio of used and new scenarios. In the lower part of the curve (Figure 2), a relatively large gain in reliability corresponds to a moderate increase in costs. Without losing much reliability, we could have saved $50 per candidate by employing twelve single rater stations. If we were contented with a reproducibility of 70%, we could cut down costs from $495 to $380 per candidate, if we used ten stations with a single rater. To reach a reliability of 0.80, we would need 16 stations with one rater per station ($570) or twelve stations with two raters per station ($605).

## Discussion

With the modifications to our MMI procedure we accomplished a gain in systematic candidate variability, a reduction in costs per candidate, and a substantial improvement of the operational procedure.

### Reliability

Generally, our results from variance component analysis are in line with findings of Eva et al. [12] and Dodson et al. [11]. The amount of variability due to systematic differences in candidate performance only accounts for roughly one sixth of overall variability. This is by far less than the candidate*rater effect, which reflects a bias in the ratings. Stations are not consistently found to be hard or easy, as the variance proportion attributable to the station effect is small. They seem to tap different attributes that candidates cope differently well with. This is reflected by the strong candidate*station effect. This interaction effect was similarly strong in the studies cited above. However, Eva et al. [12] found larger differences in rater stringency as compared to our study and a smaller candidate*station effect. This might result from more homogeneity in their stations.

From station statistics we conclude that raters used the full range of scores more unreservedly in 2010. The rise in overall reliability is due to an increase in candidate variability with a simultaneous drop in rater – and, therefore, unwanted – variance. The rise of overall rater agreement in 2010 might be due to the more elaborated training, the increased number of practice runs, and detailed verbal anchors along the rating scale. However, as we do not have a systematic variation to the procedure, we can only assume which actions for the betterment were worthwhile and the reasons for the rise in reliability remain a matter of speculation.

The reuse of stations saved time and money, but the revision did not lead to consistent amelioration in terms of inter-rater agreement or item-total correlations. There seems to be a lot of randomness in rater behavior which is reflected in the large rater*candidate term. Mediocre inter-rater agreement might partly be due to the mixed rater teams [12]. In future analyses, however, the allocation of mixed teams allows us to look for systematic differences in ratings with respect to raters’ gender and profession.

Notwithstanding, we seem to have dropped those stations that did not contribute to a reliable discrimination of candidates’ performance.

### Costs

Lower costs in 2010 were mainly due to the reuse of stations, especially test runs, as we did not need trial runs for reused stations, and the economization of station development. The disadvantage of using scenarios in subsequent years lies in the risk of provoking stereotype behavior of candidates due to their preparation for known scenarios. A large pool of interchangeable scenarios which is continually extended might be a way to reduce costs and to minimize specifically trained behavior. Even though we were able to substantially reduce the costs of our procedure, costs were still much higher as compared to those reported by other study centers. It is striking that we seem to invest a comparatively large amount of money into station development. We estimated an approximate amount of 40 staff hours for the development, testing and revision of one scenario and the corresponding score sheet as opposed to three hours as allocated by Rosenfeld et al. [14]. In our study, we report all costs involved, including working hours by volunteers within our faculty who take part in the development process. At our university the raters highly value elaborated scenarios and score sheets. This might be due to a general resistance to unstructured interviews. Interviews are not commonly used for admission decisions in Germany.

### Cost-effectiveness

To increase overall reliability, it is more cost-efficient to raise the number of stations instead of raising the number of raters within stations. However, there may be limitations to the number of stations that can practicably be employed. For instance, the limited number of adjacent rooms available might force officials to restrict the number of stations used. It is generally worthwhile to adjust for rater stringency, as this reduces error and improves reliability without producing further costs.

With our 2010 procedure, we could have saved costs of $10,000 (or $50 per candidate) while approximately maintaining overall reliability if we had used twelve stations with a single rater. If we had been looking for a more cost-efficient, time-saving procedure with a minimum of 70% reproducibility, the most feasible way would have been the employment of ten single rater stations which would have saved a total of $23,000. Moreover, it is important to note that we granted interviewers the comfort of not having to give ratings. While costs could be reduced by including interviewers in the rating process ($55 per candidate or $11,000 in total), we cannot say how this would affect the reliability of the procedure.

We still face the unresolved question of validity: We reduced the number of target variables but still we do not know what we are measuring. Inter item correlations, item-total correlations, and inter-rater reliabilities were generally low as has been observed in other studies, and the high candidate*station effect suggests that we tap different skills and characteristics that are individually distinct in candidates. This was the case for both, the 2009 and the 2010 run. This is a typical bandwidth-fidelity dilemma.

### Strengths and limitations of the study

This study evaluated the HAM-Int procedure under a high stakes condition. This, however, entails that we have a nested design. We have two raters at each station, and therefore, we can estimate inter-rater reliabilities for each station. It would be interesting to disentangle our error term. The rater*candidate interaction reflects a bias we want to eliminate from our procedure. It is difficult to say which actions have led to the better results in 2010. Another limitation of our study is the difference between the candidate samples. The 2009 sample was very heterogeneous as candidates were only preselected by GPA. Candidates were completely unprepared as MMIs were not at all common in Germany, and candidates received no information about the procedure beforehand. In 2010, candidates had more information and had the chance to prepare themselves for the interviews. The 2010 cohort was more homogeneous due to the pre-selection by HAM-Nat and GPA.

### Outlook

As costs are still very high, we aimed to reduce rater hours. In the following years, we developed written tasks and used advanced students as observers. Score sheets are continually revised and feedback of raters included. The MMI needs to be developed further to reduce overall costs and to ensure reliable scores. Another task will be to tackle the validity of the procedure. In the subsequent years we included self-report measures to investigate external validity.

## Conclusion

With our reorganization of the procedure we achieved a gain in reliability as well as a reduction in costs. Still, the costs of our MMI are much higher than the costs of written tests. Because of the fixed costs associated with the procedure, it is worthwhile to test a large number of candidates. We need to invest in station development, and we need to reduce unsystematic variance due to rater behavior, because an increase in reliability by adding more stations or raters is dearly paid for in the top reliability scores.

## Abbreviations

GPA: Grade point average; HAM-Int: Hamburg assessment test for medicine – interview; HAM-Nat: Hamburg assessment test for medicine – natural sciences; MMI: Multiple mini - interview.

## Competing interests

The authors have no conflicts of interests.

## Authors’ contributions

SS and JH performed the statistical analysis, and JH drafted the manuscript. WH and SH conceived of the study, and participated in its design and coordination and helped to draft the manuscript. All authors read and approved the final manuscript.

## Authors’ information

Johanna Hissbach is a psychologist and research fellow in the admission testing team at the Medical Faculty of Hamburg University, Germany.

Susanne Sehner is a research fellow at the Department of Medical Biometry and Epidemiology at the Medical Faculty of Hamburg University, Germany.

Sigrid Harendza, MD, MME, is professor for Internal Medicine and Educational Research and Development at the Medical Faculty of Hamburg University, Germany.

Wolfgang Hampe is professor for biochemistry with the focus on teaching. His team develops and realizes the admission procedure for Hamburg Medical School.

## Pre-publication history

The pre-publication history for this paper can be accessed here:

http://www.biomedcentral.com/1472-6920/14/54/prepub
